# Determinants of occupational injuries among building construction workers in Kampala City, Uganda

**DOI:** 10.1186/s12889-019-7799-5

**Published:** 2019-11-04

**Authors:** Arthur Kiconco, Nathan Ruhinda, Abdullah Ali Halage, Stephen Watya, William Bazeyo, John C. Ssempebwa, Joseph Byonanebye

**Affiliations:** 1 Makerere University School of Public Health, P. O. Box 7072, Kampala, Uganda; 2 Marquette University, Milwaukee, USA

**Keywords:** Occupational injuries, Linear models, Perception, Safety, Construction workers, Workplace

## Abstract

**Background:**

Globally, about 1000 people die and close to 860,000 people sustain injury at work daily. Injury prevention and control require contextual evidence, although most studies in Uganda have focused on general causes. Factors associated with occupational injuries among building construction workers were assessed in this study.

**Methods:**

A cross-sectional study among building construction workers was conducted in Kampala, Uganda. A standardized semi-structured questionnaire was used to collect data. Three hundred nineteen (319) participants were randomly and proportionately selected from 57 construction sites. Descriptive statistics were used to describe the variables while generalized linear modeling was used to estimate the crude/adjusted prevalence ratios.

**Results:**

The prevalence of occupational injuries was 32.4%. Most injuries, approximately 70% occurred among nightshift workers. Age of ≤24 years (APR: 2.09 CI: 1.20–3.65, *P* = 0.009); daily income in or above the second quartile−USD ≥3.2 (APR: 1.72, CI: 1.06–2.80, *P* = 0.028); job dissatisfaction (APR: 1.63, CI: 1.17–2.27, *P* = 0.004); job stress (APR: 1.72, CI: 1.22–2.41, *P* = 0.004); poor safety environment (APR: 1.51, CI: 1.10–2.05, *P* = 0.009); PPE provision (APR: 1.47, CI: 1.05–2.05, *P* = 0.02) and routine use of PPE (APR: 0.57, CI: 0.34–0.95, *P* = 0.03) were significantly associated with occupational injuries.

**Conclusion:**

There was a relatively high prevalence of injuries mostly resulting from cuts and mostly suffered on night duty. Upper and lower extremities were the most hurt parts of the body during injury leading to loss of a substantial number of productive days. This could affect the health and wellbeing of construction workers. Most of the factors significantly associated with occupational injuries are modifiable thus an opportunity to address the problem. Efforts towards integrating education for behaviour change, advocacy and training workers to demand for their rights to safe and protection at work and legislation enforcement can help reduce occupational injury occurrence.

## Background

Occupational injuries are a public health and economic problem globally with about 5–7% of all fatalities in industrial countries attributable to work-related injuries [1]. In low-income countries, non-occupational health problems pose a bigger burden, but work-related injuries also pose a substantial burden [2]. The International Labour Organization (ILO) estimates that 860,000 people sustain injury or ill health at work daily, and nearly 1000 people die worldwide as a result of occupational injuries daily [3]. In 2010 alone, over 313 million suffered non-fatal injuries at work globally leading to at least 4 days of absence from work [3]. Every year, 350,000 deaths are due to fatal occupational injuries; but 270 million serious non-fatal injuries also occur [4].

Construction industry is more risky [5, 6] as compared to other industries due the high burden of occupational hazards [7, 8]. Building construction workers are three to four times more likely to be killed and twice as likely to be injured compared to workers in other occupations [9, 10]. The rate of the injuries is low in high-income countries than in middle and low-income countries (LMICs) despite the low rate of reporting of occupational injuries in LMICs. It is estimated that more than 16,000 fatal occupational injuries occur in the established market economies with a fatality rate of 4.2 and accident rate of 3240 per 100,000 workers respectively. These are lower than the fatality and accident rates in developing countries like in Asia at 21.5 and 16,434 per 100,000 workers respectively and Sub-Saharan Africa at 21 and 16,012 per 100,000 workers respectively [11].

In 2013, occupational injuries in Tanzania were estimated at 36 fatal injuries per 1000 workers—a fatality rate of 23.73 per 100,000 workers [12]. Rwanda reported a total of in 482 non-fatal accidents among 130 respondents in a 780 man-months period construction industry in 2012 and building construction projects were found to have higher accident rates compared to other civil engineering projects [13].

In Uganda, work-related injuries continue to be a big problem and the situation of the building construction industry is considered one of the most dangerous [14]. Reports from Uganda’s Ministry of Gender, Labour and Social Development (MoGLSD) show that injuries among construction workers accounted for 13% of all occupational injuries in Kampala in 2003 and were the third contributor of injury events [15]. Over 60% of all occupational accidents in the country occur in Kampala [16] and the injury and fatality rate for Kampala district stood at 4248 per 100,000 and 92 per 100,000 workers respectively in 2014 [17]. Uganda just as other low-income countries is on the struggle to meet population needs. Building construction is one of the fast growing industries in Kampala city characterized by frequent accidents [14]. Occurrence of occupational injuries at construction sites is as a result of complex interactions between individuals and the work environment; however, proximate probable risk factors include poor service and maintenance of construction equipment, inadequate training of workers, and congestion on building sites [17]; inadequate supervision, poor quality materials, psychological problems and inadequate lighting for night shifts workers [14].

Several measures to prevent and control occupational injuries in Uganda include Occupational Safety and Health (OSH) Act of 2006, which calls for employers to protect their workers by ensuring that all possible measures to ensure that workers and public are free from danger at workplaces, however, occupational injuries continue to claim lives at construction sites. Contextual evidence is required for successful injury prevention and control although most studies in Uganda have focused on general causes rather than association of different factors with occupational injury. Therefore, our study explored the socio-demographic, work environment and behavioral factors associated with occupational injuries in order to inform relevant injury prevention and control efforts.

## Methods

### Study design and period

A descriptive cross-sectional study in which quantitative data was collected was conducted between April and May 2016.

### Study setting

The study was carried out in Kampala City–Uganda’s capital city located in Central Uganda between April and May 2016. Kampala district in general has a population of 1,516,210 people, at a 2.02% growth rate per annum since 2002 [18]. As the population grows, infrastructural development in Kampala is also fast compared to other urban areas in Uganda.

### Study population

The study involved building construction workers aged 18 years of age and above and were actively engaged in the industry during the study period.

### Eligibility

Building construction workers of 18 years of age and above who had worked in the construction industry for at least 6 months prior to the study and were still actively engaged in the construction industry and available at construction sites during the study period were recruited for the study. Workers who had disabilities that could not allow them to fully express their views in regard to the study questions were not involved in the study.

### Sample size

Using Kish Leslie (1964) formula for cross-sectional studies and prevalence of occupational injury at 27% [19], a sampling error (δ) of 5% at 95% confidence level, a minimum sample of 303 respondents was computed. A response rate of over 99.5% has been reported in similar studies [20, 21], therefore, a non-response rate of 5% was considered thus a final sample size of 319 respondents.

### Sampling procedure

Simple random sampling with sampling proportionate to size was used. Lists of registered building sites were obtained from Department of Physical Planning at Kampala Capital City Authority (KCCA) before making the general list for sampling. A total of 455 building construction sites across the five divisions of Kampala City were considered. Sites in each division were assigned computer generated random numbers and 12% were selected resulting in 57 sites.

To select workers, a list of all workers at each site including supervisors was obtained from the foreman after approval from the site manager and each worker was assigned a number. A number of workers proportionate to the number of workers at respective sites was randomly selected. In cases where a selected respondent declined to participate, the next number on the list was considered.

### Data collection tools and procedure

Face to face interviews using a questionnaire with close ended and semi-structured questions were used to collect the data. Trained research assistants visited construction sites, making appointments with the selected respondents and then interviewed them at appropriate times and in privacy.

### Variable measurement

To assess injury prevalence, we asked about history of occupational injury within 6 months prior to the study that led to at least 6 hours off the job. To assess factors including poor safety of the work environment, workplace supervision, risk taking behaviour, job satisfaction and job stress; a validated three-scale item standardized worker’s response device (WRD) questionnaire [22] was adapted with adjustments to fit in Uganda’s context supplemented with Jenkins four item sleep scale [23] to assess sleeping disorder. For the WRD, factors were measured by summing scores of individual items under each factor. The 90th percentiles of the scores for each factor was used as threshold value where scores <90th percentile were taken as poor and those ≥90th percentile were taken as good [20, 22].

### Data analysis

Data was analysed using Stata® 12.0. At univariate analysis frequencies and percentages were used to describe the distribution of respondents in each of the study variables. The association of each of the independent variables was assessed at bivariate analysis using generalized linear modelling with modified Poisson regression. Crude Prevalence Ratios (cPRs) at 95% confidence interval were calculated. Multivariate analysis of all variables with *p*-value ≤ 0.20 to adjust for confounding to generate adjusted Prevalence Ratios (APRs) and effect modification was assessed. All statistical tests were two-sided, 95% confidence intervals were used and *p*-values of ≤0.05 were considered statistically significant.

### Quality control

One day training of research assistants was conducted to ensure quality data collection. The training covered question clarity, interview skills as well as appropriate information capturing. The questionnaire was pretested on 10 participants at a site that was later excluded in the study and then adjusted in respect to the pretesting results to ensure validity of the questions.

## Results

A total of 318 respondents from 58 construction sites were interviewed. The response rate of this study was 99.7%, and only one respondent did not complete the questionnaire.

### Socio-demographic characteristics and injury prevalence among construction workers

**Fig. 1:**
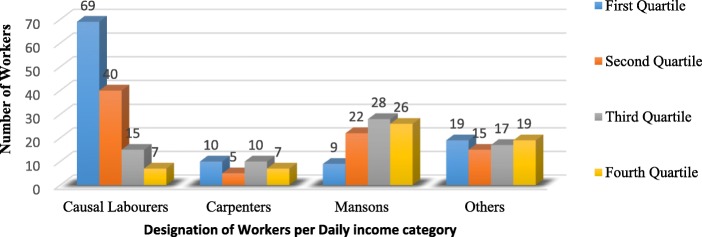
Distribution of construction workers by designation and experience

**Fig. 2:**
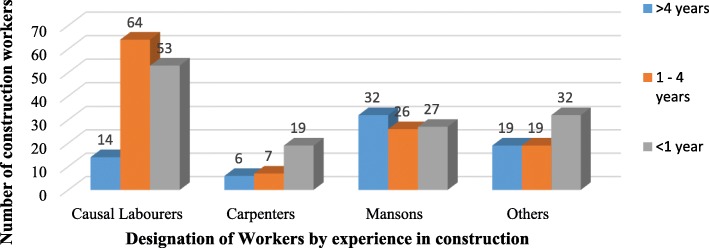
Distribution of construction workers by designation and working hours

**Fig. 3:**
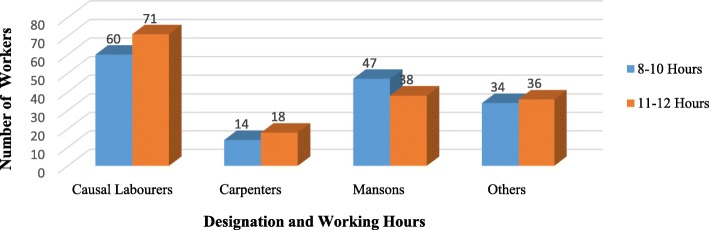
Distribution of construction workers by designation and daily income

Table 1 shows the socio-demographic characteristics of the respondents. Majority were aged 25–35 with age range 18–57 years, mean age 28.2 and standard deviation (SD) 7.0. Most (90.9%) of the respondents were males. Close to a half (47.2%) had completed secondary school and majorly (72.6%) temporary workers. Daily income ranged from 1.9–2.7USD (first quartile), 3.2–4.0USD (second), 4.9–5.4USD (third quartile) to ≥6.8USD (fourth quartile) with majority (33.65%) in the first quartile. Majority (72.6%) of the workers did not have contracts with their employers and the biggest percentage (63.5%) had spent more than a year in building construction work with majority of causal labourers having experience of 1-4 years (Fig. 1). Slightly more than a half (51.2%) especially causal labourers (Fig. 2) worked for more than 10 h a day yet the daily income of 59.43% of the workers was in the second quartile and below especially the causal labourers whose daily income fell in the first quartile (Fig. 3). Injury prevalence was higher among those below 35 years, males, temporary employed, worked for over 10 h and those whose daily income fell in the upper 2 quarters (Table 1).

**Table 1:** Socio-demographic Characteristics and Injury Prevalence among Construction Workers in Kampala, Uganda

Variables	Injury	
	Yes n (%)	No n (%)
Age (years)		
35+	11 (22.0)	39 (79.0)
≤ 24	42 (36.52)	73 (63.48)
25–35	50 (32.68)	103 (67.32)
Sex		
Female	6 (20.69)	23 (79.31)
Male	97 (33.56)	192 (66.44)
Marital status		
Single	54 (35.06)	100 (64.94)
Married	45 (29.61)	107 (70.39)
Separated/Widowed	4 (33.33)	8 (66.67)
Highest Education Level		
Tertiary	28 (34.15)	54 (65.85)
Primary	24 (30.77)	54 (69.23)
Secondary	49 (32.67)	101 (67.33)
None	2 (25.00)	6 (75.00)
Employment Terms		
Contract	25 (28.74)	62 (71.26)
Temporary	78 (33.77)	153 (66.23)
Experience in building Construction		
1–4 years	28 (24.14)	88 (75.86)
< 1 year	44 (33.59)	87 (66.41)
> 4 years	31 (43.66)	40 (56.34)
Working hours		
8–10	44 (28.39)	111 (71.61)
11–12	59 (36.20)	104 (63.80)
Daily income (Quartiles)		
First	24 (23.3)	83 (38.60)
Second	18 (17.48)	64 (29.77)
Third	32 (31.07)	38 (17.67)
Fourth	29 (28.16)	30 (13.95)

### Characteristics of workers who had suffered an occupational injury

Table 2 shows characteristics of workers who had occupational injuries. The prevalence of occupational injuries was 32.4%. The majority (68.9%) of the respondents suffered injury while on night duty. Of those who experienced injuries, 27.2% were cut by sharp objects. The majority (70.9%) sought medical care after the injury and close to a half (46.6%) went back to work in less than 1 day.

**Table 2:** Prevalence and characteristics of occupational injury among construction workers in Kampala, Uganda

Variables	Frequency (*n* = 318)	Percent (%)
Had an injury at work in the last 6 months		
Yes	103	32.4
No	215	67.6
What led to injury		
Fell from height	16	15.5
Pierced by construction materials	19	18.4
Cut by sharp object z	28	27.2
Hit by falling object	18	17.5
Others^a^	22	21.4
Part of the body hurt		
Hand	26	25.2
Foot	21	20.4
Leg	19	18.5
Others^b^	37	35.9
Sought medical care following the injury		
Yes	73	70.9
No	30	29.1
Admitted after injury		
Yes	14	13.6
No	89	86.4
Work shift involved in at the time of injury		
Day	32	31.1
Night	71	68.9
Time taken to go back to work		
≤ 1 day	48	46.6
2–3 days	19	18.4
> 4 days	36	35.0

^a^Electrocution, Fell at the same level, Held between objects, Hit by colleague

^b^Head/neck, shoulder, Chest, Eye, Back, Abdomen

### Distribution of respondents by work environment characteristics and injury prevalence

As indicated in Table 3, majority (41.2%) of the respondents were casual labourers (porters) and over a half (52.2%) never had any health and safety training. Even those who had ever had health and safety training, majority 50.7% (77/152) were trained at their workplaces. Over 80% were on sites without written safety and health policy/program and onsite hazard communication measures. More than quarter (31.45%) of the workers were not provided with PPE by employers and had to purchase on their own. Injuries were most prevalent among causal labourers and those without safety training.

**Table 3:** Work Environment Characteristics among construction workers in Kampala, Uganda

Variables	Injury	
	Yes n (%)	No n (%)
Work designation		
Carpenters	12 (37.50)	20 (62.50)
Casual labourers	30 (22.90)	101 (77.10)
Masons	35 (41.18)	50 (58.82)
Others^a^	26 (37.14)	44 (62.86)
Ever had health and safety training		
Yes	45 (29.61)	107 (70.39)
No	58 (34.94)	108 (65.06)
Trained from		
Training Institute	18 (24.66)	55 (75.34)
Workplace	23 (29.11)	56 (70.89)
Written health and safety policy/program		
Yes	18 (28.57)	45 (71.43)
No	85 (33.33)	170 (66.67)
On site hazard communication measures		
No	10 (43.48)	13 (56.52)
Yes	93 (31.53)	202 (68.47)
Workplace Supervision		
Good	87 (32.22)	183 (67.78)
Poor	16 (33.33)	32 (66.67)
PPE Provision		
Employer	60 (27.52)	158 (72.48)
Worker	43 (43.00)	57 (57.00)
Poor safety environment		
No	55 (26.19)	155 (73.81)
Yes	48 (44.44)	60 (55.56)

Others^a^ = Designers, electricians, equipment operators, supervisors, cooks, guards, plumbers and trainees

### Behavioural characteristics of the workers and injury prevalence

Among construction workers who drank alcohol, only 31.5% (29/92) drunk before going to work and only 34.8% (32/92) drunk while at work (Table 4). Only 10.1% (33/318) chewed khat, of whom 60.6% (20) chewed khat before going to work. More than a quarter (30.2%) experienced sleeping disorder, 24.1% (77/318) experienced job dissatisfaction, 51.8% (165/318) had job stress, and 25.2% (80/318) exhibited risk-taking behaviour. More than a quarter (100/318) of the respondent provided PPE to themselves and only 32.7% (104/318) always used PPE (Table 4).

**Table 4:** Behavioural Factors and Injury Prevalence among Construction Workers in Kampala, Uganda

Variables	Injury	
	Yes n (%)	No n (%)
Alcohol Drinking		
No	76 (33.63)	150 (66.37)
Yes	27 (29.35)	65 (70.65)
Type of alcohol taken		
Local	21 (27.71)	56 (72.73)
Packaged	2 (33.33)	4 (66.67)
Drink alcohol before work		
No	17 (28.81)	42 (71.19)
Yes	8 (27.59)	21 (72.41)
Drink alcohol at work		
No	20 (34.48)	38 (65.52)
Yes	5 (19.23)	21 (80.77)
Khat Chewing		
No	95 (33.33)	190 (66.67)
Yes	8 (24.24)	25 (75.76)
Chew khat before going to work		
No	6 (30.0)	14 (70.00)
Yes	3 (37.5)	5 (62.50)
Chew khat at work		
No	6 (37.5)	10 (62.50)
Yes	3 (25.0)	9 (75.00)
Cigarette Smoking		
No	91 (32.73)	187 (67.27)
Yes	12 (30.00)	28 (70.00)
Kind of cigarettes smoked		
Industrial	8 (36.36)	14 (63.64)
Local	3 (23.08)	10 (76.92)
Sleeping disorder		
No	63 (65.63)	152 (68.47)
Yes	33 (34.38)	70 (31.53)
Job dissatisfaction		
No	36 (46.75)	174 (72.20)
Yes	67 (27.80)	41 (53.25)
Job stress		
No	35 (22.88)	97 (58.79)
Yes	68 (41.21)	118 (77.12)
Risk taking behaviour		
No	71 (29.83)	167 (70.17)
Yes	32 (40.00)	48 (60.00)
Use of PPE		
Always	36 (34.62)	68 (65.38)
Sometimes	52 (35.37)	95 (64.63)
Never	15 (22.39)	52 (77.61)

### Factors associated with occupational injuries

Table 5 shows the factors that are associated with occupational injuries after adjusting for all other factors. Age of ≤24 years (APR: 2.09 CI: 1.20–3.65, *P* = 0.009); daily income in or above the second quartile−USD ≥3.2 (APR: 1.72, CI: 1.06–2.80, *P* = 0.028); job dissatisfaction (APR: 1.63, CI: 1.17–2.27, *P* = 0.004); job stress (APR: 1.72, CI: 1.22–2.41, *P* = 0.004); poor safety environment (APR: 1.51, CI: 1.10–2.05, *P* = 0.009); PPE provision (APR: 1.47, CI: 1.05–2.05, *P* = 0.02) and routine use of PPE (APR: 0.57, CI: 0.34–0.95, *P* = 0.03) were significantly associated with occupational injuries.

**Table 5:** Factors associated with occupational injuries among building construction workers in Kampala, Uganda

Variables	Injury		cPR [95% CI]	*P*-value	aPR[95% CI]	*P*-value
	Yes	No				
Work designation						
Carpenters	12 (37.50)	20 (62.50)	1		1	
Causal labourers	30 (22.90)	101 (77.10)	0.61 [0.37–1.05]	0.07	0.62 [0.38–1.01]	0.66
Masons	35 (41.18)	50 (58.82)	1.09 [0.65–1.83]	0.72	0.80 [0.50–1.28]	0.36
Others^†^	26 (37.14)	44 (62.86)	0.99 [0.57–1.70]	0.97	1.19 [0.71–1.77]	0.59
Sex						
Female	6 (20.69)	23 (79.31)	1		1	
Male	7 (33.56)	192 (66.44)	1.62 [0. 78–3.37]	0.195	1.19 [0.61–2.35]	0.60
Highest Education Level						
Tertiary	28 (34.15)	54 (65.85)	1		1	
Primary	24 (30.77)	54 (69.23)	0.90 [0.57–1.41]	0.30	1.05 [0.67–1.64]	0.82
Secondary	49 (32.67)	101 (67.33)	0.95 [0.65–1.39]	0.71	1.15 [0.79–1.66]	0.44
None	2 (25.00)	6 (75.00)	0.73 [0.21–2.53]	0.21	1.42 [0.57–3.54]	0.45
Age						
35+	11 (22.0)	39 (79.0)	1		1	
≤ 24	42 (36.52)	73 (63.48)	1.66 [0.93–2.95]	0.17	2.09 [1.20–3.65]	0.009*
25–34	50 (32.68)	103 (67.32)	1.48 [0.84–2.63]	0.08	1.68 [0.97–2.89]	0.06
Employment Terms						
Contract	25 (28.74)	62 (71.26)	1		1	
Temporary	78 (33.77)	153 (66.23)	1.17 [0.80–1.71]	0.402	0.91 [0.63–1.32]	0.63
Experience in building Construction						
1–4 years	28 (24.14)	88 (75.86)	1		1	
< 1 year	44 (33.59)	87 (66.41)	1.39 [0.93–2.08]	0.11	1.14 [0.78–1.64]	0.49
> 4 years	31 (43.66)	40 (56.34)	1.81 [1.19–2.74]	0.005*	1.37 [0.91–2.07]	0.13
Ever had health and safety training						
Yes	45 (29.61)	107 (70.39)	1		1	
No	58 (34.94)	108 (65.06)	1.18 [0.85–1.62]	0.31	1.20 [0.88–1.64]	0.24
Working hours						
8–10	44 (28.39)	111 (71.61)	1		1	
11–12	59 (36.20)	104 (63.80)	1.27 [0.92–1.76]	0.14	1.05 [0.77–1.44]	0.73
Daily income (Quartiles)						
First	24 (23.3)	83 (38.60)	1		1	
Second	18 (17.48)	64 (29.77)	0.98 [0.57–1.67]	0.93	0.91 [0.54–1.51]	0.75
Third	32 (31.07)	38 (17.67)	2.03 [1.31–3.15]	0.001*	1.72 [1.06–2.80]	0.028*
Fourth	29 (28.16)	30 (13.95)	2.19 [1.41–3.19]	< 0.001*	1.72 [1.07–2.77]	0.025*
Job dissatisfaction						
No	36 (46.75)	174 (72.20)	1		1	
Yes	67 (27.80)	41 (53.25)	1.68 [1.23–2.30]	0.001*	1.63 [1.17–2.27]	0.004*
Job stress						
No	35 (22.88)	97 (58.79)	1		1	
Yes	68 (41.21)	118 (77.12)	1.80 [1.27–2.54]	0.001*	1.72 [1.22–2.41]	0.002*
Poor safety environment						
No	55 (26.19)	155 (73.81)	1		1	
Yes	48 (44.44)	60 (55.56)	1.69 [1.24–2.31]	0.001*	1.51 [1.10–2.05]	0.009*
Risk taking behaviour						
No	71 (29.83)	167 (70.17)	1		1	
Yes	32 (40.00)	48 (60.00)	1.34 [0.96–1.86]	0.08	1.24 [0.87–1.78]	0.22
PPE Provision						
Employer	60 (27.52)	158 (72.48)	1		1	
Workers	43 (43.00)	57 (57.00)	1.56 [1.14–2.13]	0.005*	1.47 [1.05–2.05]	0.02*
Use of PPE						
Always	36 (34.62)	68 (65.38)	1		1	
Sometimes	15 (22.39)	52 (77.61)	0.64 [0.38–1.08]	0.10	0.57 [0.34–0.95]	0.03*
Never	52 (35.37)	95 (64.63)	1.02 [0.72–1.44]	0.90	0.78 [0.55–1.10]	0.16

*Significant association; *cPR* crude Prevalence Ratio, *aPR* adjusted crude Prevalence Ratio; Others^†^ = Designers, electricians, equipment operators, supervisors, cooks, guards, plumbers and trainees

## Discussion

This study describes the characteristics and determinants of occupational injuries among building construction workers in Kampala City. We explored the prevalence and the association of socio-demographic, work environment and behavioural factors with occupational injuries. There was a high prevalence of occupational injuries of 32% among workers. This prevalence is not surprising since many construction sites did not have onsite hazard-communication measures, written safety and health policy, and majority did not use personal protective equipment (PPE). A higher prevalence has been reported elsewhere, 46.2% in Mit-Ghamr [24] and 38.7% in Gondar [21] cities of Egypt, as well as 38.3% [25] in Ethiopia. This study also shows that the biggest percentage of workers had suffered injury while on night duty. This is in line with Dembe, et al. [26] who reported extended working hours and another study that reported inadequate lighting for night shifts [14] as predictors for occupational injuries. Non-use of PPE calls for efforts towards behaviour change among workers while neglected safety and health to promote safety at the workplaces should be observed.

In this study, over a quarter of those who experienced injuries were cut by sharp objects, while the rest were pierced by construction materials/equipment, hit by falling objects, fell from heights, electric shocks, fell at the same level, held between objects or hit by colleagues. These are among the fatal four as described by ILO [27] and the United States Occupational Safety & Health Administration [28]. Similar results have also been reported by many scholars including falls [10, 17, 24, 29], electrocution [10] being struck by falling objects [10, 17] being hit by machinery and hand tools [17, 20, 30]; and cutting edges [17, 19]. Majority of the respondents got injuries on their hands, feet, legs or head/neck, shoulder, chest, eye, back or abdomen. This indicates that personal protective equipment targeting extremities and other safe working practices would make a change in building construction. These findings are in line with others who reported injuries to the upper and lower limbs [24], upper trunk and extremities, i.e. eyes, neck, back, shoulder, arm, finger, and hand [31]. Loss of productive time is an important issue as far as occupational injuries are concerned and this study reveals that over a half of those who suffered injuries could only get back to work after two to four or more days. Just as it is reported that non-fatal occupational injuries led to at least 4 days of absence from work in 2010 [3]. In Denmark, the mean days of work lost due to injury was 3.21 in 2013 which lead to an estimated 1,822,000 workdays which were about 6% of the total absence from work due to all types of illness [32]. This poses a big burden on both the health system and families [27] as besides men being the majority yet most of the times breadwinners in most homes, they may also require care during the time of nursing the injuries. It has been noted that in many cases, other family members may have to forfeit their jobs so as to care for an injured worker [33] and in the US, 2007 estimates indicate that the direct and indirect cost of work injuries was $192 billion [34]. Indeed, an earlier study had indicated that the cost of home care of injured family members by other household members was about 6.2 million workdays a year an equivalent of $162 million [35]. In addition to that, in an economy where majority of the workers are men, on temporary recruitment, and not covered by any kind of insurance, it means that all the costs of the injuries maybe borne by the injured workers or their families which over burdens the families.

Long experience in building construction work did not show significant association with occupational injuries and illness in this study. Many other studies [36, 37] have however reported association of long job experience and injury occurrence. Chau and colleagues [20] reported work experience less than 5 years to be associated with injuries, but Bena and others [38] in their study found that injury rates decreased with increase in job tenure with high risk among those who had worked for less than 6 months and risk reduction after 2 years of work experience. Other studies have reported that experience less than 1 year [39, 40] increases the risk of injuries. Just as noted in other literature [25] besides lack of use of PPE as well as other safety promotion measures at different sites, high prevalence among the experienced may be due to the fact that experienced workers may get used to the working environment and take safety measures for granted thus exposure to hazards and increased risk of injury.

We also found that prevalence of injury among workers whose daily income was in the third and fourth quartile (≥3.2 USD) was 1.72 times than that among those whose daily income was the first or second quartile. This implies that the prevalence of accidents was lower among workers who earn less which is contrary to the findings by other scholars [41, 42] who reported high risk of occupational injuries among workers with low income levels. Though not plausible in Ugandan literature, authors mention that wages always increase as workplace risks increase [43]. We may also speculate that high-risk tasks could have been offered at a high pay to attract workers’ interest thus increased exposure and the observed high injury prevalence. It was also observed that the prevalence of injury was higher among workers with more than 4 years of experience. People spend more time in their workplace than even their homes, hence workplaces become part of their life yet it involves many hazards thus high risk for occupational injuries. Construction workers even most of the times live away from their homes and in substandard accommodation [27]. Although many studies have indicated that extended working hours per day or week [21, 24, 26], safety and health training of construction workers reduce occupational injuries [37, 44, 45], these factors were not statistically significantly associated with injury occurrence in this study.

Among respondents who perceived that their work environment was not safe, the prevalence on injury was 1.5 times higher as compared to those who perceived that their work environment was safe. Other scholars [21, 24, 46] have also indicated that poor perception of workplace safety is a predictor of workplace injury although a promoter of safety culture at the same time [46] arguing that workers with positive perception of their workplace safety are less likely to report accidents as well as a lower number of self-reported injuries compared to those with negative perception [24]. Although competent supervision is reported to promote safety and health in workplaces [24, 26, 47], in this study, it was not significantly associated with high prevalence of injury among workers. This may be because most of the workers reported good supervision at their workplaces hence significant differences could not be detected.

Consistent with other studies, this study indicated that the prevalence of occupational injuries was 1.63 times among workers who were dissatisfied with their job as compared to those who were satisfied. This is in line with Osman and Kumie [48] who found that workers who were dissatisfied with their assigned jobs were 1.8 times more likely to be injured compared to those who were satisfied. This may be attributed to the fact that satisfaction affects workers’ commitment to workplace procedures and directions, which may increase their risks for occupational injury. Being about a balance between employee’s objectives and those of the organization, job satisfaction becomes such a controversial issue to understand and deal with from either sides. Dissatisfied workers may find no meaning and reason to take responsibility or focus on safety precautions which may exacerbate their risk for injury [21].

Workers with job stress were about two times more likely to experience injuries compared to those who never had job stress. Job stress is a complex issue but its association with injury experience may be due to both negative perception of the work environment, working at unbearable speeds or for long periods as well as need to follow appropriate procedures thus lack of concentration on safety precaution and consequently the increased injury risk. The findings of this study support other studies that have also identified stress as an important risk factor for occupational injury [24, 36, 49–51]. Stress may be due to work burden, repetitive work, and job uncertainty [52] thus complicated tasks may lead to job stress which through many pathways may lead to injury, for, example a person stressed due to fear of falling [27] and perceived poor work environment [21] may end up getting an injury due to operation with little confidence.

Khat chewing was not associated with building construction accidents in this study yet it has been found to be a risk factor for occupational injury in other studies especially when there is addiction [20, 48]. This could have been due to the small number of workers found to chew khat while at work thus losing power for comparison. The small number of workers could have been due to under reporting because of social desirability bias, as some workers may not admit chewing khat at work because of their prior knowledge that it is a negative behaviour.

The prevalence of injury was 1.47 times among those who were not provided with PPE by their employers compared to their counterparts who were provided with PPE. This could be attributed to the fact that majority of workers earned low income, which may limit their capacity to purchase, own and use PPE. However, use of PPE has been reported to reduce occurrence of occupational injuries [25, 53].

### Limitations

This study did not go without limitations. Recall bias among respondents was a potential problem since the questions required 6 months recall of past events of injury. In this regard, respondents who sustained serious injuries could have easily remembered the injuries compared to those who experienced minor injuries. Social desirability could have occurred because of the questions that required one to answer behavioural and practices. Underrepresentation could have occurred since the study only considered registered construction sites; unregistered construction sites may have been missed. However, the findings of this study can be generalised beyond registered sites since workers do not differ much and registration does not necessary come with safety inspections.

## Conclusion

There was a relatively high prevalence of injuries mostly resulting from cuts and mostly suffered on night duty. Upper and lower extremities were the most hurt parts of the body during injury leading to loss of a substantial number of productive days. Daily income above the second quartile, job dissatisfaction, job stress, PPE provision and use and poor safety environment were significantly associated with occupational injuries. This could affect the health and wellbeing of construction workers. Occupational injury prevention and control measures, which include approaches integrating education for behaviour change, advocacy and training workers to demand for their rights to safety and protection at work, and legislation enforcement should be implemented in Uganda. Finally, more analytical studies should be conducted to inform control and prevention efforts in Uganda.

## Data Availability

All the significant data supporting our findings is contained within the manuscript. However, all supplementary datasets analysed during the current study are available from the corresponding author on reasonable request.

## Abbreviations

APRs: Adjusted Prevalence Ratios
APWM: Accidents per working month
cPRs: crude Prevalence Ratios
ILO: International Labour Organisation
KCCA: Kampala Capital City Authority
LMICs: Middle and low-income countries
MoGLSD: Ministry of Gender, Labour and Social Development
OSH: Occupational Safety and Health
PPE: Personal Protective Equipment
USA: United States America
USD: United States Dollar
